# Image-guided cochlear access by non-invasive registration: a cadaveric feasibility study

**DOI:** 10.1038/s41598-020-75530-7

**Published:** 2020-10-27

**Authors:** Jiang Wang, Hongsheng Liu, Jia Ke, Lei Hu, Shaoxing Zhang, Biao Yang, Shilong Sun, Na Guo, Furong Ma

**Affiliations:** 1 Department of Otorhinolaryngology - Head and Neck Surgery, Peking University Third Hospital, Peking University, No. 49 North Garden Road, Haidian District, Beijing, 100191 China; 2 The Robotics Institute, School of Mechanical Engineering and Automation, Beihang University, Beijing, China

**Keywords:** Diseases, Therapeutics

## Abstract

Image-guided cochlear implant surgery is expected to reduce volume of mastoidectomy, accelerate recovery, and improve safety. The purpose of this study was to investigate the safety and effectiveness of image-guided cochlear implant surgery by a non-invasive registration method, in a cadaveric study. We developed a visual positioning frame that can utilize the maxillary dentition as a registration tool and completed the tunnels experiment on 5 cadaver specimens (8 cases in total). The accuracy of the entry point and the target point were 0.471 ± 0.276 mm and 0.671 ± 0.268 mm, respectively. The shortest distance from the margin of the tunnel to the facial nerve and the ossicular chain were 0.790 ± 0.709 mm and 1.960 ± 0.630 mm, respectively. All facial nerves, tympanic membranes, and ossicular chains were completely preserved. Using this approach, high accuracy was achieved in this preliminary study, suggesting that the non-invasive registration method can meet the accuracy requirements for cochlear implant surgery. Based on the above accuracy, we speculate that our method can also be applied to neurosurgery, orbitofacial surgery, lateral skull base surgery, and anterior skull base surgery with satisfactory accuracy.

## Introduction

Image-guided technology is currently widely used in surgical fields, such as neurosurgery and hepatobiliary surgery, and allows surgeons to determine the boundaries of important anatomical structures and surgical paths^1,2^. Surgical robotic systems have the advantages of good tremor filtration, mechanical repeatability, and high stability, and can compensate for the physiological disadvantages of humans, and have consequently gained attention in the past 2 decades^3^. However, image-guided surgical technology and robotic systems have found limited clinical application in otology.

Cochlear implantation (CI) is the most effective treatment for sensorineural hearing loss. To date, more than 300,000 patients have received cochlear implant surgery worldwide and have benefited markedly from this treatment^4^. During this operation, the otologist not only spends much time and energy to complete mastoidectomy, but also needs to avoid vital anatomical structures that are hidden inside the temporal bone, such as the facial nerve, chorda tympani nerve, ossicular chain, sigmoid sinus, etc. The complex anatomy makes CI surgery well-suited for image-guided and robotic assistance. Therefore, minimally invasive surgery for CI has gained interest.

In recent years, some scholars have proposed the concept of minimally invasive tunnels, with a diameter slightly larger than the electrode array of the CI, drilled into the cochlea from the mastoid cortex. After cochleostomy, the electrode array is inserted into the cochlea along this trajectory. To drill the linear path, two devices were invented: a micro-stereoscopic frame^5–7^ and robotic arm^8,9^. Labadie et al.^5–7^ designed an individualized micro-stereoscopic frame that can be anchored around the patient's temporal bone, which not only secures the drill bits while drilling, but also defines the surface markers for registration, with an accuracy of 0.38 mm in a cadaver experiment^10^. Currently, the micro-stereoscopic frame system has not only completed extensive verification in ex-vivo experiments, but has also been used for clinical patients^11^. Additionally, novel channel approaches such as the round window (RW) access^12^ and updated technological solutions such as revision operation for a device failure^13^ have recently been reported. However, designing such an individualized micro-stereoscopic framework takes at least a few days^6,7^ and causes additional trauma to the patient's skull. Bell et al. replaced the prepositioning frame with an industrial robot arm, which can hold the drill bit and is fixed to the robot base^8,9^. Improvements in the robotic system achieved an accuracy of 0.15 ± 0.08 mm for the cochlear target point in ex-vitro experiments^8^. In 2017, this research group first reported the clinical experience of using a surgical robot system to achieve the path drilling for cochlear implantation, assessed its safety, and described the surgical workflow^14,15^. Obviously, both drilling methods have been taken clinical application.

Accuracy of the robotic system is essential to the success of cochlear implant surgery. The distance between the facial nerve and the chorda tympani nerve is about 2.4–5.7 mm^16,17^, and the width between the edge of the drill and the facial nerve should be approximately 0.5 mm or less due to allow the insertion of the electrode array^14^. Additionally, it should be noted that the heat generated during drilling might damage the facial nerve. Labadie et al.^11^ reported a case of facial nerve palsy due to thermal damage by a robotic surgery. Although there is no clear value for the safety distance between the drill and the facial nerve, both research groups believe that maintaining a sufficient distance is part of the safety mechanism of a robot assisted cochlear implantation^11,14,15^. Therefore, some reports presented that the accuracy of the surgery as a whole should be less than 0.5 mm^15,18^. Generally, sources of error in the surgery include visual tracking error of the navigation system, scanning instrument error, robotic system error, and registration error, etc.^19^ At present, it is difficult to reduce the errors from hardware facilities such as the navigation system and robotic system due to the limitations of the industrial technology. However, the registration error can reduce by choosing different registration tools and registration algorithms^19,20^.

There are many methods for registration, including invasive registration methods, such as bone anchor registration, and non-invasive registration methods, such as oral fixed-reference frames, anatomical landmarks, and skin-adhesive positioning markers^21^. However, the accuracy of non-invasive registration methods is too low to meet the surgical requirements of cochlear implants. Therefore, the most accurate registration tool is a bone anchor, which involves implanting several screws into the surface of the skull as a reference mark for path planning and intraoperative navigation^21^. Bone anchors increase the patient's surgical trauma and the likelihood of infection, which is not beneficial to postoperative rehabilitation. Consequently, there is a need for a non-invasive registration method that meets the accuracy requirements for CI.

We proposed a non-invasive registration method that involves a visual-positioning frame for connecting the maxillary dentition based on an oral reference fixture. A custom-designed robotic system was used to complete the accuracy verification of a temporal bone model under the visual navigation system. Subsequently, we applied this in image-guided, minimally invasive CI on 5 cadaveric temporal specimens to verify the safety and feasibility of using this robotic system and non-invasive registration method for CI.

## Results

**Figure 1:**
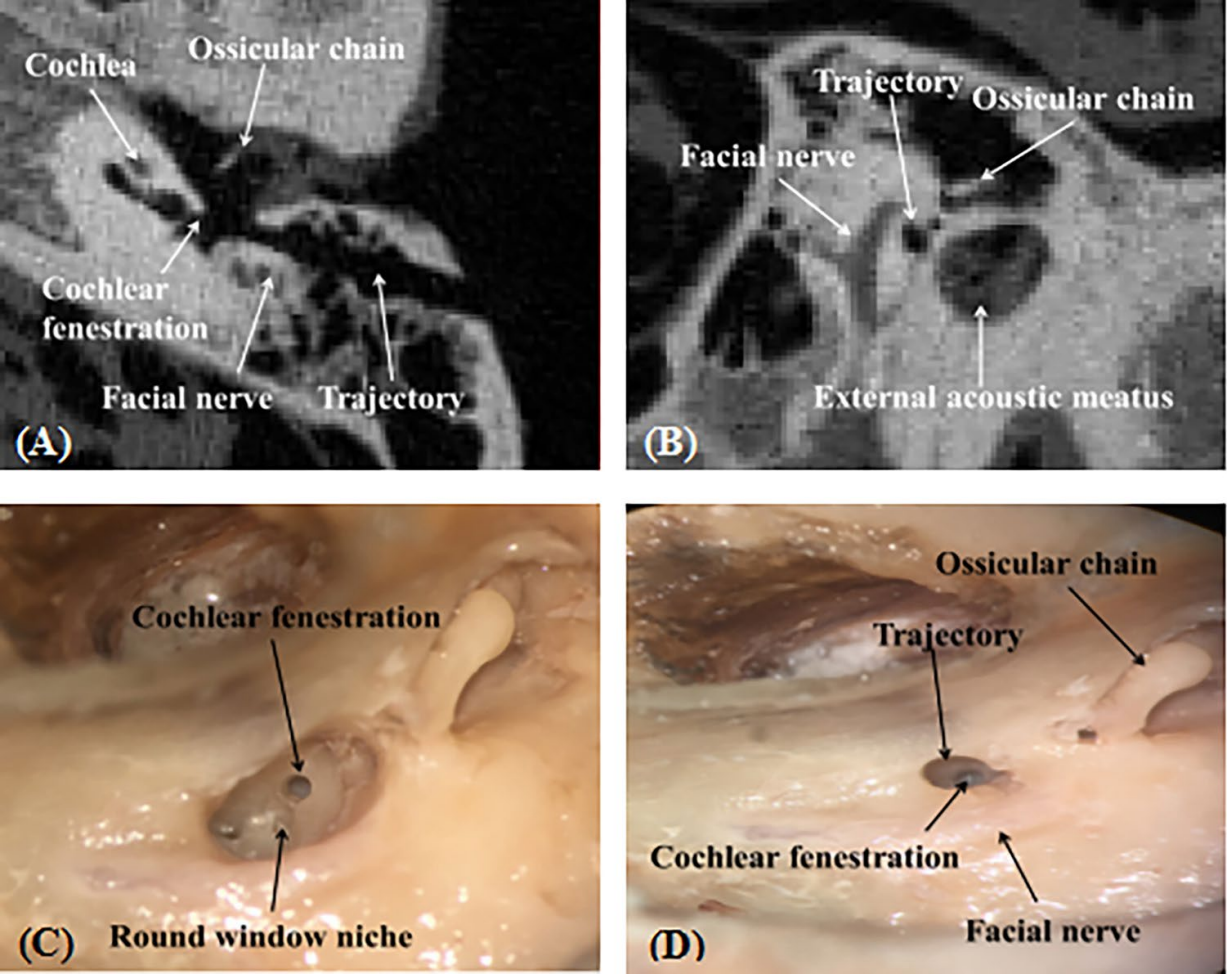
(**A**, **B**) The location between the trajectory and surrounding anatomic structures in high-resolution computed tomography. (**C**) The location relationship between the drill path and facial nerve in routine temporal anatomy. (**D**) The target point is on the basal turn of the cochlea.

In this study, mastoidectomy was replaced with a tunnel approach to allow minimal removal of the mastoid bone and significant reduction of the incision. Using the robotic system, we achieved 8 planned trajectories successfully by non-invasive registration, and in all specimens, cochleostomies were completed on the basal turn of the cochlea (Fig. 1). In three cases, preoperative images showed that facial recesses were extremely narrow; hence, the chorda tympani had to be sacrificed during the path planning. Additionally, the chorda tympani in one case and external auditory canal in two cases were damaged during the drilling. Facial nerves, tympanic membranes, and ossicular chains were perfectly preserved in all cases.

After surgery, we reconstructed the important structures and surgical paths of the 8 cases. The average length of the whole trajectory was 25.084 ± 3.311 mm, while the angular error between the drilled and planned path was 1.148 ± 0.395°. The maximum and minimum deflection angles of trajectory were 1.772° and 0.375°, respectively. The average errors of the skull entry point and the cochlear target point were 0.471 ± 0.276 mm and 0.671 ± 0.268 mm, respectively. The maximum error of target point was 1.177 mm while the minimum was 0.318 mm. The errors of the entry point were between 0.147 and 1.086 mm in all 8 cases. In addition, we calculated that the average shortest distances from the margin of the surgical tunnels to the external auditory canal and the ossicular chains were 0.976 ± 0.278 mm and 1.960 ± 0.630 mm, respectively. The shortest distances of the facial nerve, our primary concern, from the margin of the trajectory was between 0.094–2.264 mm. The statistical values from all cases are compiled in Table 1.

**Table 1:** Summary statistics from the drilling tests (n = 8).

ID	Errors (mm)		Distance (mm)			Trajectory length (mm)	Angle (degrees)
	Entry	Target	FN	EAC	AOC		
1L	0.368	0.854	2.264	0.504	1.869	26.608	1.053
1R	0.147	0.708	0.191	-	2.763	25.469	1.374
2L	0.401	0.530	0.554	0.842	1.236	22.206	1.278
2R	0.397	0.318	1.356	-	1.410	21.307	0.375
3L	0.589	1.177	0.094	1.324	2.884	32.078	1.772
4L	1.086	0.714	0.561	1.108	1.491	25.005	1.238
4R	0.427	0.418	0.779	1.044	1.678	24.459	1.069
5L	0.355	0.648	0.517	1.036	2.346	23.536	1.021
AVG	0.471	0.671	0.790	0.976	1.960	25.084	1.148
SD	0.276	0.268	0.709	0.278	0.630	3.311	0.395

*FN* Facial Nerve, *EAC* External Auditory Canal, *AOC* Auditory Ossicular Chain, *SD* standard deviation.

**Figure 2:**
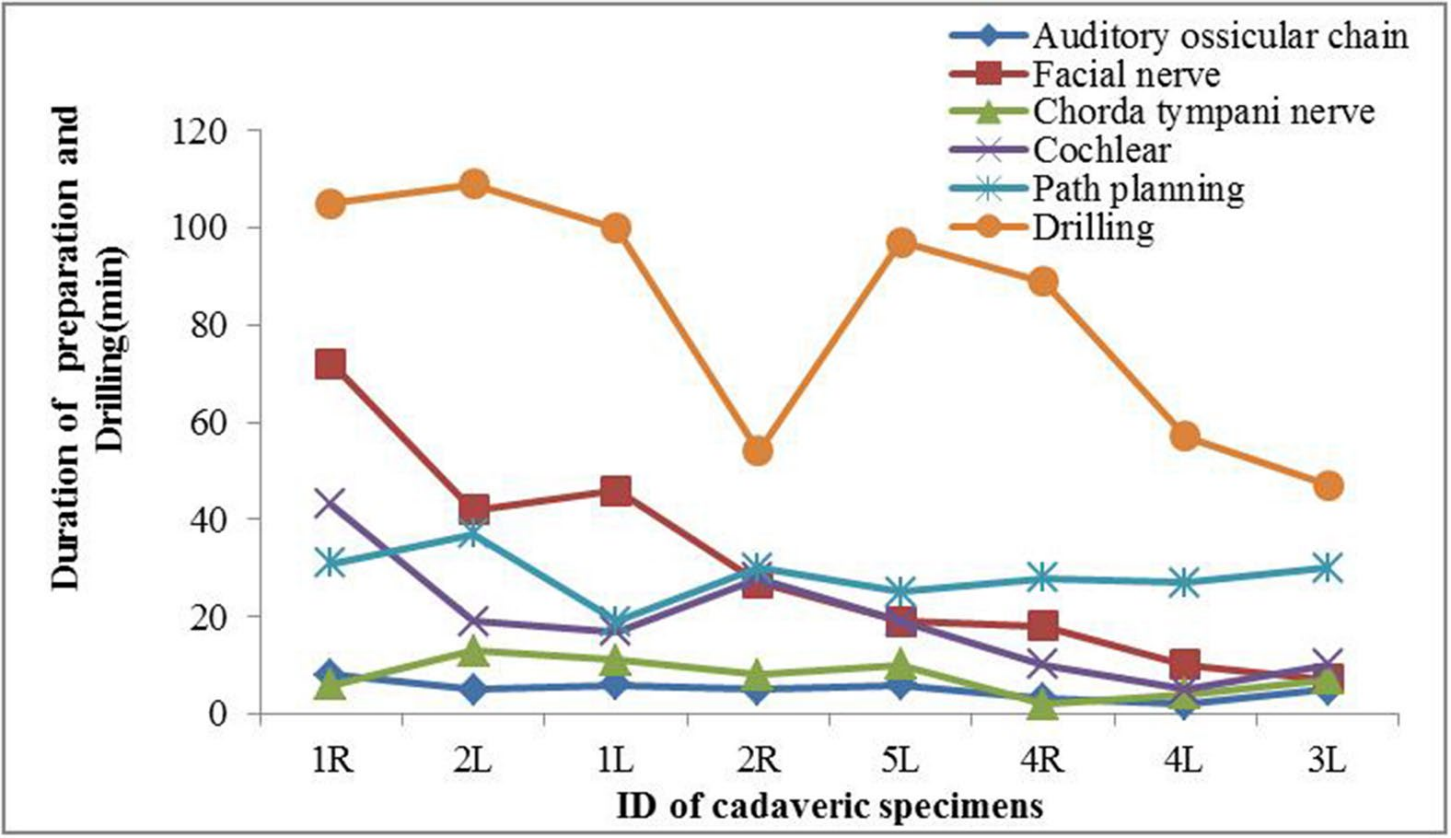
Operation duration curve of eight cadaveric heads. The preoperatively segmented structures include facial nerve, chorda tympani, cochlea, auditory ossicular chain, etc.

In Fig. 2, we show the operation time of each step such as segmentation, planning, and drilling during the image-guided cochlear implantation procedures. The total operation time of 8 specimens was 165.125 ± 57.110 min while the durations of segmentation, path planning, and drilling were 61.625 ± 35.480 min, 28.375 ± 5.181 min, 82.25 ± 25.325 min, respectively. With proficient practice and procedures, the operation duration of each step was reduced significantly, and the total time of the last operation was approximately 105 min. In all procedures, the drilling process consumed the longest time. The duration of both segmentation and path planning was within 30 min for the last specimen. The steps for registering and fixing the robot were run parallel to segment important structures and path planning.

## Discussion

This study aimed to design a non-invasive tool for registration, using a robotic system to complete the trajectory drilling from the skull surface to the cochlea under the image-guiding system. Moreover, we calculated the accuracy of the whole surgical robotic system and concluded that robot-assisted minimally invasive CI surgery was feasible by non-invasive registration.

Registration is the key to image-guided surgery, to ensure that it corresponds with the preoperative CT data and the actual spatial position in the patient intraoperatively^21^. To date, only 2 methods could achieve this goal: artificial addition of fiducial markers on the skull, and use of the patients’ own anatomical landmarks^22^. The more fixed the relative position between the registration mark and the skull, the higher the accuracy. Since the patients’ own anatomical landmarks are difficult to recognize by the navigation system, registration has mostly been achieved with the aid of an external reference mark. Currently, the micro-stereotactic positioning frame and titanium screws are the tools used for image-guided cochlear implant surgery^7,8^. However, these methods of registration increase the risk of injury and infection to the patients. Therefore, we previously sought to use the characteristics around the bone bed used in CI and the patient's own short process of the malleus in a modified, hybrid registration, which has an accuracy of 0.86 mm^23^. Although such accuracy is not satisfactory, for relatively large important structures in the deep temporal bone, such as the internal carotid artery or the jugular bulb (which have relatively low accuracy requirements), this method was sufficient.

Upper jaw, as a part of the skull, is one of the most stable bones, and maxillary dentition can be exposed to provide an anchoring foundation^24,25^. Because the spatial relationship between the upper jaw, the maxillary dentition and the temporal bone is constant, it will not be displaced by external factors such as changes in the patient’s postures, edema, etc^5,24–26^.Therefore, bony markers, such as the upper teeth, can be used to fix fiducial marks around the mastoid, which is not only safe and non-invasive, but is also easy to disinfect and install before surgery^24,27^. Eggers et al.^27^ placed titanium screws on the molars and the premolars, and measured accuracy at the level of the lateral skull base of 3.31 mm. Although such accuracy was unsatisfactory, considering that the CT scan layer thickness was as high as 1 mm and the position of the marks was too far from the measurement target point, such a large error was inevitable^27,28^. To reduce the error, reference markers were installed around the surgical area (mastoid) through the cantilever and the scanning layer thickness of the CT was reduced to 0.5 mm, and the registration error was, surprisingly, lowered to 0.73 ± 0.25 mm^5,24^.

Based on the results of such experiments, we hypothesized that, if the thickness of the CT layer could be further reduced, we could complete robot-assisted CI surgery under a navigation system with an independently developed robot arm. For this reason, we redesigned a non-invasive registration framework, replacing 7 screw-fits with 4 marks that are more easily recognized by the navigation system, and enveloping the skull entry points by adjusting the up and down movement of the connecting rod. To verify the accuracy of the whole system under non-invasive registration while taking into account the many factors influencing surgery, we drilled the ears of 5 corpses (8 cases). In all cases, the target points were achieved at the basal turn of the cochlea and cochleostomy was performed successfully. However, compared with the registration method of titanium fiducial markers from the research group of Bern University, the accuracy of our method was lower (less than 0.3 mm)^8,29^. The following reasons may be responsible for this phenomenon. First, high-resolution CT with a layer thickness of 0.4 mm was used in our experiment while Bell and their colleagues obtained temporal bone imaging by cone beam CT (CBCT)^8,29,30^. The CBCT has a lower radiation dose and layer thickness is approximately 0.15 mm, which can reduce the error of image-guided surgery^31^. Second, this was the first time our system had completed specimen experiments, and there are still many programs and algorithms for improvement and optimization in the whole system. Just as Bell et al.^30^ performed in vitro experiments for the first time, the target error was over 0.5 mm which improved to 0.15 mm after improving the robotic drilling system^8^. We believe that with the maturity of the system and the reduction of CT layer thickness, the error of the cochlear target point can be further reduced.

To date, some non-invasive registration tools, such as headset, skin-adhesive markers, laser surface scanning, etc., have been utilized in image-guided surgery, but these have been less accurate^32–39^. The main reasons for this lack of accuracy may be that these fiducial systems are not firmly connected to the skull and are far from the operation area, which affects the recognition of the navigation system. Compared to these less invasive registration methods, we achieved a higher accuracy with a method that was easier to operate (Table 2).

**Table 2:** Target accuracy of different non-invasive registration methods.

Registration method	Invasive or not	Target registration error/target point error	Application
Bone‑bed and the short process of malleus^23^	Minimal invasive	0.30–1.29 mm	Cochlear Implantation
Laser Surface Scanning^32^	Non-invasive	1.25 ± 0.64 mm	Neurosurgery
Headset^33^	Non-invasive	1.44 ± 0.24 mm	Lateral skull base surgery
Headband^34^	Non-invasive	1.46 ± 0.15 mm	Lateral skull base surgery
Granular jamming cap^34^	Non-invasive	0.56–1.40 mm	Skull base surgery
LED mask^35^	Non-invasive	0.92 ± 0.13 mm	Sinus and skull base surgery
Skin adhesive markers^36^	Non-invasive	2.49 ± 1.07 mm	Neurosurgery
Anatomical landmarks^37^	Non-invasive	0.93 ± 0.31 mm	Orthognathic surgery
Dental splint^38^	Non-invasive	0.55 ± 0.28 mm	Frontolateral skull base surgery
Template-assisted marker positioning^39^	Non-invasive	1.2 ± 0.12 mm	Temporal bone surgery
Our study	Non-invasive	0.671 ± 0.268 mm	Cochlear Implantation

Compared with traditional cochlear implant surgery, keyhole insertion of electrode array faces greater challenges due to limitations of the visual operative field and operating space^40^. Two types of insertion tools including manual^40,41^ and automatic^42,43^ insertions have been reported for the new method of accessing the cochlea through the tunnel. Currently, these implantation tools have been implemented clinically and have achieved satisfactory performance^11^. Generally, surgeons are mainly considered with whether the electrode array was accurately inserted into the Scala and the risks of intra- cochlear trauma, which is closely related to the preoperative path planning (insertion angle) and the performance of the insertion tool. In addition, the risks of tip fold-over and the damage of electrode array in the implantation progress is higher than traditional surgery^11^. Therefore, the design of special electrodes for image-guided cochlear implant surgery and the improvement of the insertion tools performance are the highlight in future research of our group. In our experiment, the cochleostomy target points in some cases were not in the plane of scala tympani due to the existence of target error. Currently, the advantages of the RW approach have been fully verified^44^, and some studies have been reported that electrode arrays were inserted through the RW access^12,45^. Therefore, our team has begun to implement robotic drilling plan that toward the RW, and complete electrode insertion through RW with the assistance of implantation tools or manually.

For experienced surgeons, the whole time of traditional cochlear implantation is about 135 min while the surgical duration is approximately 80–90 minutes^46^. However, in the clinical implementation that has been reported, the total time of image-guided cochlear implant surgery was longer than 90 minutes^11,12,14^, which was widely divergent with the original assumption (less than 50 min)^47^. In our study, the additional time except for drilling was very similar to the results reported by Caversaccio et al.^12^. In recent years, some researchers have proposed new methods for automatic segmentation of important structures in the temporal bone and automatic planning of trajectory, which can reduce the preoperative preparation time to several minutes^48,49^. Currently, our research group is testing another method that use the neural network based on deep learning to perform automatic segmentation and path planning in temporal bone CT.

Some issues need to be highlighted. First, frames interference with the intubation process was considered but the nasotracheal intubation can be used in during the general anesthesia process of the cochlear implant surgery. Second, whether the fixation using silicone rubber will remain stiff enough during the procedure has to be considered. On the one hand, the weight of the frame is light enough (only 70 g) that is almost impossible dislocated and loosened from maxillary dentition. On the other hand, the occlusion between the upper and lower dentition helps to fix the frame to a certain extent. Previous studies have demonstrated that this non-invasive frame was rigid and repeatable^5,27,50^. In the previous research of our research group, we measured the deviation of the relative position after repeatedly wearing the navigation frame. The average displacement error was 0.03 mm and the maximum angular deviation was less than 0.07°, which further indicated that our navigation frame has extremely high stability and repeatability.

Image-guided technology is also a vital method for skull base surgery, as this anatomic region is deep and narrow, and contains critical structures^51^. For example, the cavernous sinus is surrounded by cranial nerves, including the abducens nerve, the oculomotor nerve, the trochlear nerve, and the third, fourth, and sixth divisions of trigeminal nerves^52^. Recently, biopsy of the cavernous sinus through the foramen ovale has become a minimally invasive diagnostic method and requires an accuracy of less than 1.0 mm^53^. Several tumors, including schwannoma, cavernous angioma, skull base meningioma, hypophysoma, and cerebellopontine angle tumor, require safe and accurate drilling of the compact bone^54^; such drilling also requires planning of the surgical path with such a navigation system and avoiding important structures^55^. For the anterior skull base, a navigation system can help in locating the frontal sinus, internal carotid artery, optic nerves, etc.^56^ For lateral skull base surgery, such a system can also help to locate the internal auditory canal, jugular bulb, sigmoid sinus, major arteries, and nerves during opening of the petrous bone^54^. Several previous studies have shown that the targeting accuracy in skull base surgery must be in the sub-millimeter range to protect important structures^55,57^. Several working groups have attempted some preliminarily registration methods in skull base surgery, including screw markers (0.66 ± 0.08 mm)^35^, sole mask (0.92 ± 0.13 mm)^35^, a mapper cage (2.87 ± 0.98 mm)^21^, surface registration (1.59 ± 0.14 mm)^58^, and anatomic landmarks (4.37 ± 0.73 mm)^58^, which are invasive or imprecise. Our registration strategies could attain high precision and are non-invasive, although our methods have not performed in skull base surgery. However, our approach provides a new registration method for skull base surgery. By adjusting the structure of the visual positioning frame and improving the technical conditions, the registration precision could be increased to meet the requirements of skull base surgery.

Image-guided technology was first developed in neurosurgery and used stereotactic frames to establish a coordinate system and stabilize the head for brain surgery^1,57^, including laser ablation, tumor resection, deep brain stimulation (DBS), biopsy, aspiration, and microsurgery, etc^59–61^. The disadvantage of stereotaxy is that it is difficult to reach millimeter-level positional accuracy^62^. For lesions deep in the brain, for example, craniopharyngiomas (adjacent to the hypothalamus, pituitary, optic nerve, and important vessels), and the risk of using this technology is high^51^. In recent years, frameless technology (e.g., landmarks) and robotics have come to be used in neurosurgery^60^. However, application of these new technologies remains challenging, particularly due to the high accuracy and minimal invasiveness of neurosurgery^63^. The frame has been replaced by the use of anatomical landmarks or fiducial markers, which imposes additional trauma or yield insufficient accuracy^60^. Our visual positioning frame allows the markers to be placed closer to the neurosurgical area with a more rigid connecting rod, making the relative position between the marks and the skull more stable, and improving registration accuracy. Additionally, this visual positioning frame may be useful in image-guided orbitofacial surgery, such as trauma repair, orbital bony decompression, orbital/orbitofacial tumor resection, and lacrimal drainage surgeries^64^.

There were some limitations to the study. Our subjects were adult specimens, while a significant proportion of patients who require cochlear implantation are children. In future, we plan to apply this registration method to child specimens to verify its safety and effectiveness. Furthermore, this was an ex-vivo study, and only 8 cases were included in the experiment. At present, our visual positioning frames are only suitable for healthy adults; we have not studied its use on patients with oral deformities or loose teeth. Designing special tooth supports for these individuals should be investigated in future.

In conclusion, in this preliminary study, we verified the feasibility of using a visual registration frame as a registration tool in image-guided CI surgery and measured the surgical accuracy achieved. Moreover, this registration method can also be applied to neurosurgery and other head and neck surgery, such as lateral skull base, anterior skull base, and orbitofacial surgery, etc. In future, various types of supports should be developed to meet the special needs of individuals, and the accuracy of registration should be further improved. This study forms the basis for eventual clinical application of image-guided, minimally invasive CI surgery, neurosurgery, and other head and neck surgery with non-invasive registration.

## Materials and Methods

### Registration

**Figure 3:**
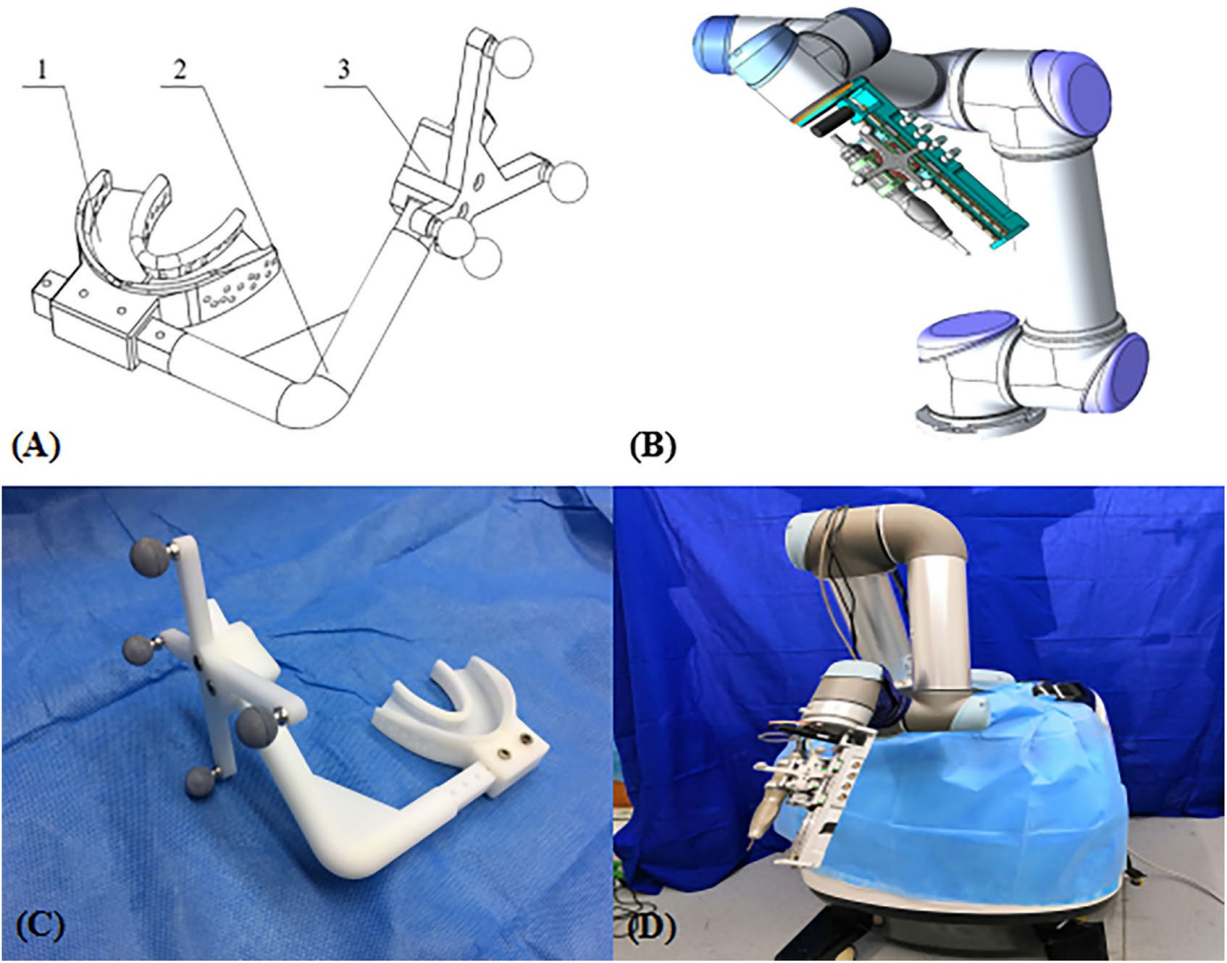
(**A**, **B**) The non-invasive registration tool, consisting of a (1) tooth support; a (2) connecting rod, and a (3) navigation frame. The Polaris stereo camera can detect the position of 4 marks on the navigation frame and then calculate the spatial position of the drill bit. (**C**, **D**) The robotic system is composed of a UR5 robot and an end effector. The bone drill handle is fixed to the end effector. Before the experiment, the robotic system is installed on 1 side of the operating platform and the direction of the arm adjusted.

We designed a non-invasive registration tool, consisting of a tooth support, a connecting rod, and a navigation frame (Navigation mark) (Fig. 3). The tooth support is tightly attached to the upper teeth by means of silicone rubber impression material, and the navigation frame is fixed around the entry point by the connecting rod. The navigation frame has 4 marks that can be recognized by the tracking system.

**Figure 4:**
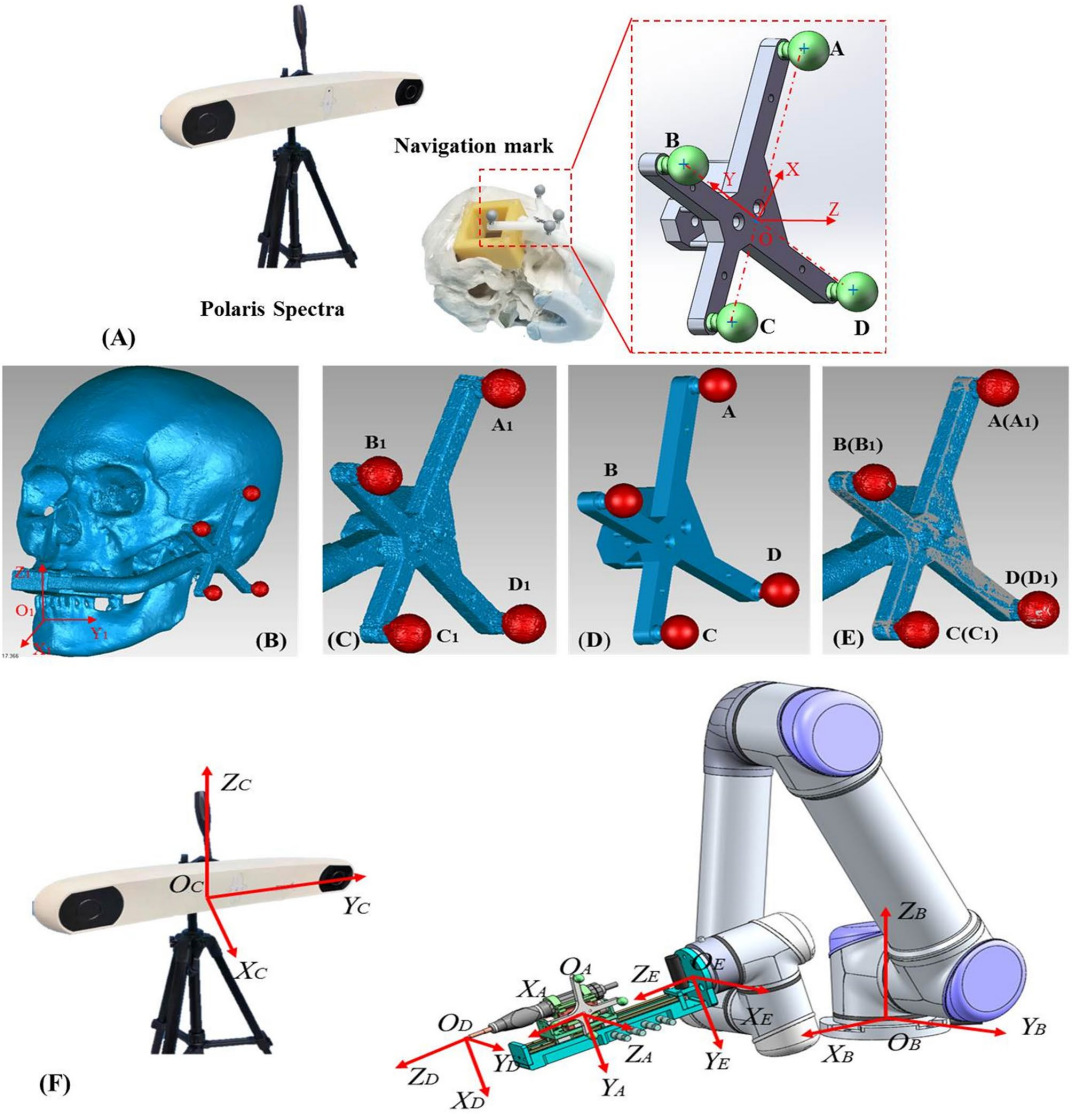
(**A**) Establish the Navigation mark coordinate system (abbreviated *{N}*) of the navigation frame in the 3D model. (**B**) Extract the Image coordinate system (abbreviated *{I}*) of the planned path and navigation frame. (**C**, **E**) Align the 2 frames and create the coordinate rotation matrix ***T*** to complete the registration. (**F**) Definition of each coordinate system of the robot system; A (*{A}*): End passive marker coordinate system; B (*{B}*): Base coordinate system; C (*{C}*): Camera coordinate system; D (*{D}*): Drilling bit coordinate system; E (*{E}*): End effector coordinate system.

We established the Navigation mark coordinate system (*{N}*) of the navigation frame in a 3D model. Then, the Navigation mark coordinate system (*{N}*) was registered in Polaris Spectra tracking system (Northern Digital Inc., Waterloo, Ontario, Canada), which was converted to pose coordinates (position and attitude information relative to the reference coordinate system) in the navigation space. In addition, we read the image coordinate system (*{I}*) of the planned path and the navigation frame. The frames in the 3D model and CT image were separately imported into the Geomagic studio 2013 software (Rock Hill, SC, USA, version 2013.0.1.1206, https:// www.geomagic.com). With this software, we used the best-fit alignment to align the 2 frames based on the Iterative Closest Point (ICP) algorithm through the marks of *{I}* (points A_1_, B_1_, C_1_, D_1_) and *{N}* (points A, B, C, D). Subsequently, the coordinate rotation matrix ***T*** was created and described the coordinate transformation between {*N*} and {*I*} to complete the registration (Fig. 4).

### Robotic system

The robotic system is composed of a small industrial robot (UR5, Universal Robots, Odense, Denmark) with 6 degrees of freedom (6-DOF) and an end effector (Fig. 3). It is a 6-joint, light robot (weighing 18.4 kg) with a maximum load capacity of 5 kg. The rotation range of each joint is 360 degrees, and the maximum speed of the end movement is 1 m/s. The independently developed end effector, with a 1-degree of freedom feed system, consists mainly of a linear motion unit, a rotary drilling unit, and a fixed base. The fixed base is the carrier of the entire system, supporting and linking to the UR5 robot. The linear motion unit is mounted on parallel slide rails, and the motor output shaft drives a bevel gear pair, which drives the rotary drilling unit through the screw nut to complete the feed and retreat. The bone drill handle is fixed to the rotary feed unit for the installation and rotational movement of the drill bit. Additionally, we installed special marking points on the rotary feed unit for real-time monitoring of 3D positions.

In order to ensure that the direction of movement of the robot arm is keep pace with the end effector under navigation, it is necessary to perform “hand-eye calibration” on the posture of the robot arm and the end coordinate system of the robot arm in the navigation coordinate system (Fig. 4). We preset 16 sets of fixed poses for the robot arm in the host computer and controlled the manipulator to move to the designated pose point, read the position and attitude information of robot arm and marker frame in the navigation camera in real time through the UDP (User Datagram Protocol) transmission protocol, and complete the calibration. Additionally, we had to calibrate the drilling bit under the navigation camera and align the axis of drilling bit to the planned path. Finally, we used a PID (Proportional Integral Derivative)—based position control strategy to complete the control of the robotic system.

### Software system

**Figure 5:**
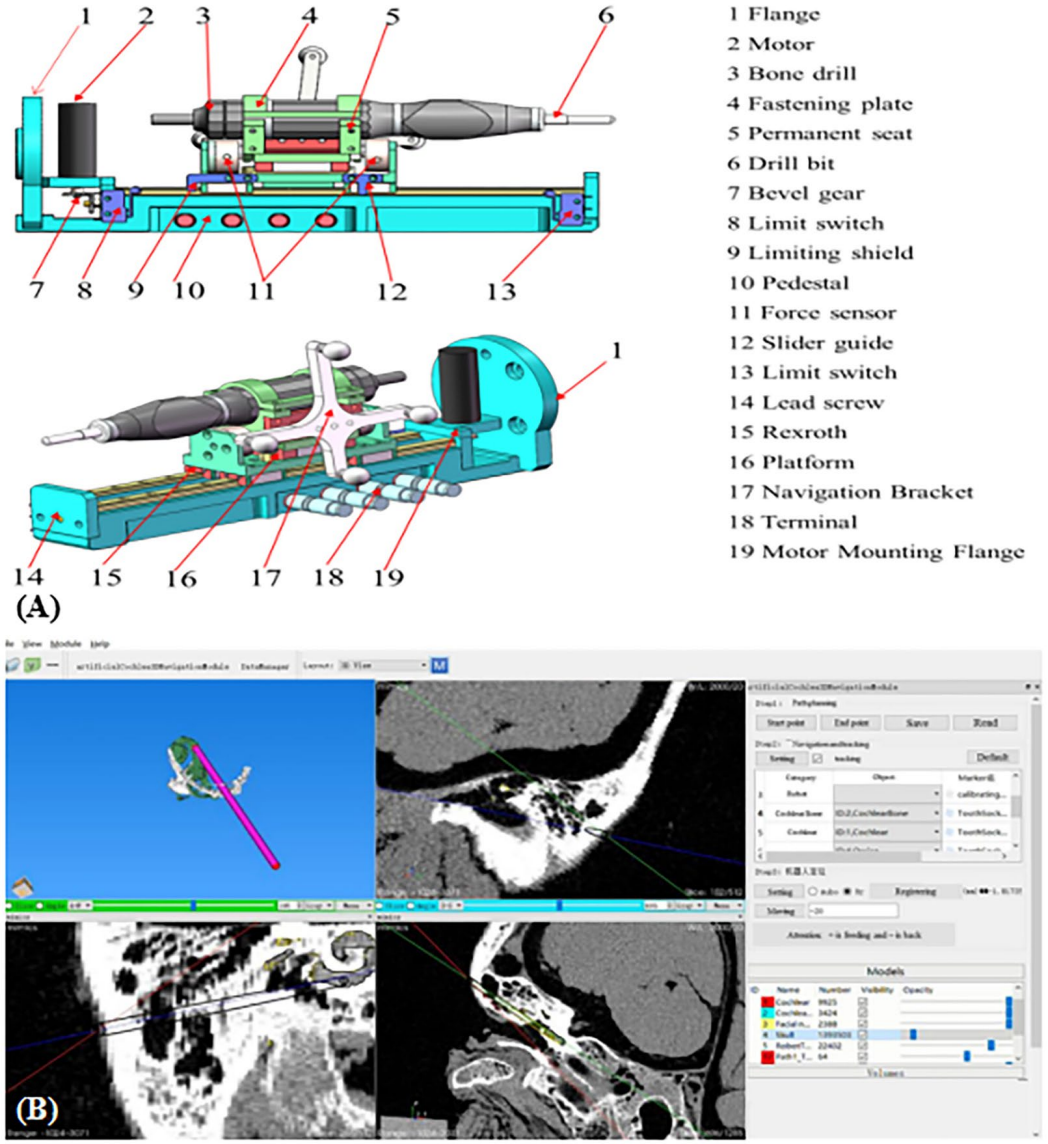
(**A**) The end effector consists of a linear motion unit, a rotary drilling unit, and a fixed base. The bone drill handle is fixed on the rotary drilling unit to achieve feed and retraction of the drill bit. (**B**) The software functions, including Toolbar (import STL data), Displaying window, Path planning, Navigation and tracking, Robot control, Models (add or remove anatomical structures and change the color and transparency of the modules), etc.

The Artificial Cochlear 3D Navigation Module system (copyright registration number: 2014SR050996, Beijing, China) was developed by the Peking University Third Hospital and Beihang University, School of Mechanical Engineering and Automation. It mainly includes modules, such as Toolbar, Displaying window, Path planning, Navigation and tracking, Robot control, and Models, which can import/delete models, modify model properties, view port rotation and switching, plan the path, perform navigation tracking, and allow robot control (Fig. 5).

### Tracking system

In this study, we used a photoelectric navigation system (Polaris Spectra, Northern Digital Inc.) to track the spatial position of the robotic arm and the specimen. The Polaris Spectra system can emit infrared light, and we installed special markers that reflect infrared light on the frame and end effector to detect the position of the drill bit in real space.

### Cadaveric temporal specimen

Five formalin-fixed adult cadaveric temporal specimens were used in this trial. The criterion for selecting specimens was that external anatomy had to be complete, without dissection deformity of the cochlea or ossicular chain, and without obvious narrowing of the facial recess. In this study, the right ears of 2 corpses were excluded due to cochlear deformity and inner ear surgery. Four titanium screws (CIBEI, Ningbo, China) were anchored in the posterior wall of the external auditory canal and mastoid zone for postoperative accuracy verification. Silicone rubber impression material was mixed with catalyst (elite HD + , Zhermack, Badia Polesine, Italy) in a 1:1 ratio; this was added to the tooth support, which was then quickly adhered to the upper teeth for firm connection with the navigation frame.

### Imaging

After registration, the specimens underwent a high-resolution 128-channel multidetector computed tomography scan (SOMATOM Definition Flash, Siemens, Munich, Germany; slice thickness = 0.4 mm, pitch = 0.2 mm) at the Department of Radiology, Peking University Third Hospital. Per our standard protocol, scanning was carried out from the lower margin of the mandible to the infraorbital margin in each specimen. Axial sections were achieved at the following settings: matrix size, 512 × 512; field of view, 220 × 220 mm; voltage, 120 kV; and current, 240 mAs. All CT images were downloaded from the physician’s workstation and saved in 512 × 512 pixels, DICOM format, for analysis.

### Structural segmentation and planning trajectory

**Figure 6:**
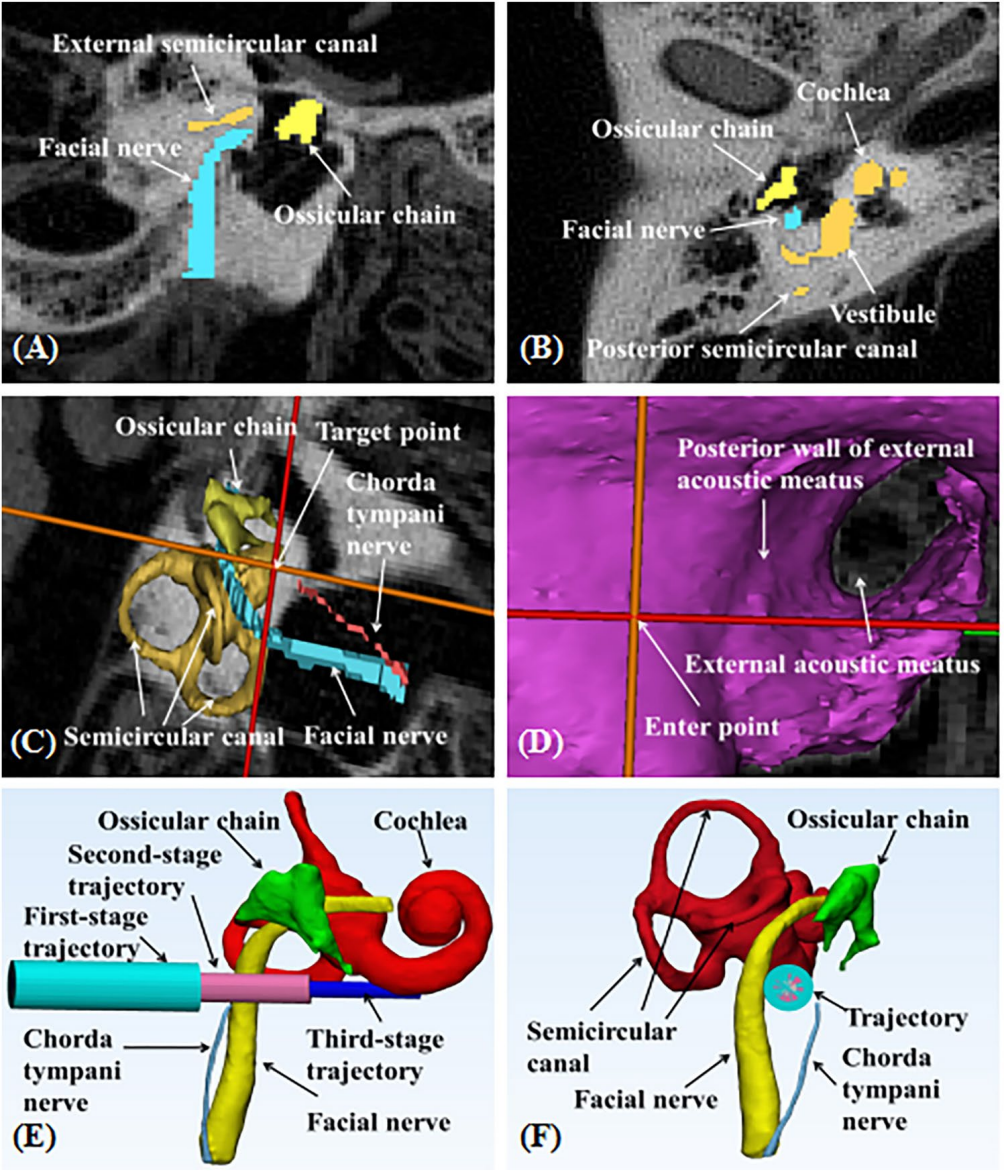
(**A**, **B**) Image segmentation of some important structures such as facial nerve, chorda tympani nerve, cochlea, Ossicular chain, etc. (**C**, **D**) Determine the coordinates of the skull entry points and cochlear target points in Mimics 20.0 software. (**E**, **F**) Correct the coordinates of the entry and target points in 3-matic software Version 12.0, then automatically generate the drilling tunnels and record the final coordinates.

Using Mimics image processing software (Materialise NV, Leuven, Belgium, version 20.0, https://www.materialise.com), we segmented the important structures from the CT data: facial nerve, chorda tympani nerve, cochlea, ossicular chain, etc. The locus at 0.5 mm anteroinferior to the RW, and a site in the mastoid area, 1 mm behind the posterior wall of the external acoustic meatus, were respectively selected as the target point and the entry point. The coordinates of these 2 points were recorded. Then, images of these structures of interest were exported from Mimics software as stereolithography (STL) files and imported into 3-matic software Version (Materialise NV, version 12.0, https://www.materialise.com). To ensure that the trajectory was sufficiently distant from the facial nerve and at a tangent to the scala tympani of cochlea, we revised the coordinates of the skull entry point and target point in the 3-matic software (Fig. 6).

### Robotic image-guided drilling

We used a 3-stage drilling method to complete the experiment. The first-stage trajectory (3.0-mm diameter) was from the skull surface to the facial recess, the second-stage trajectory (1.8-mm diameter) was through the facial recess to the middle ear cavity, and the third-stage trajectory (1.0-mm diameter) was from the middle ear cavity to the cochlear target area.

**Figure 7:**
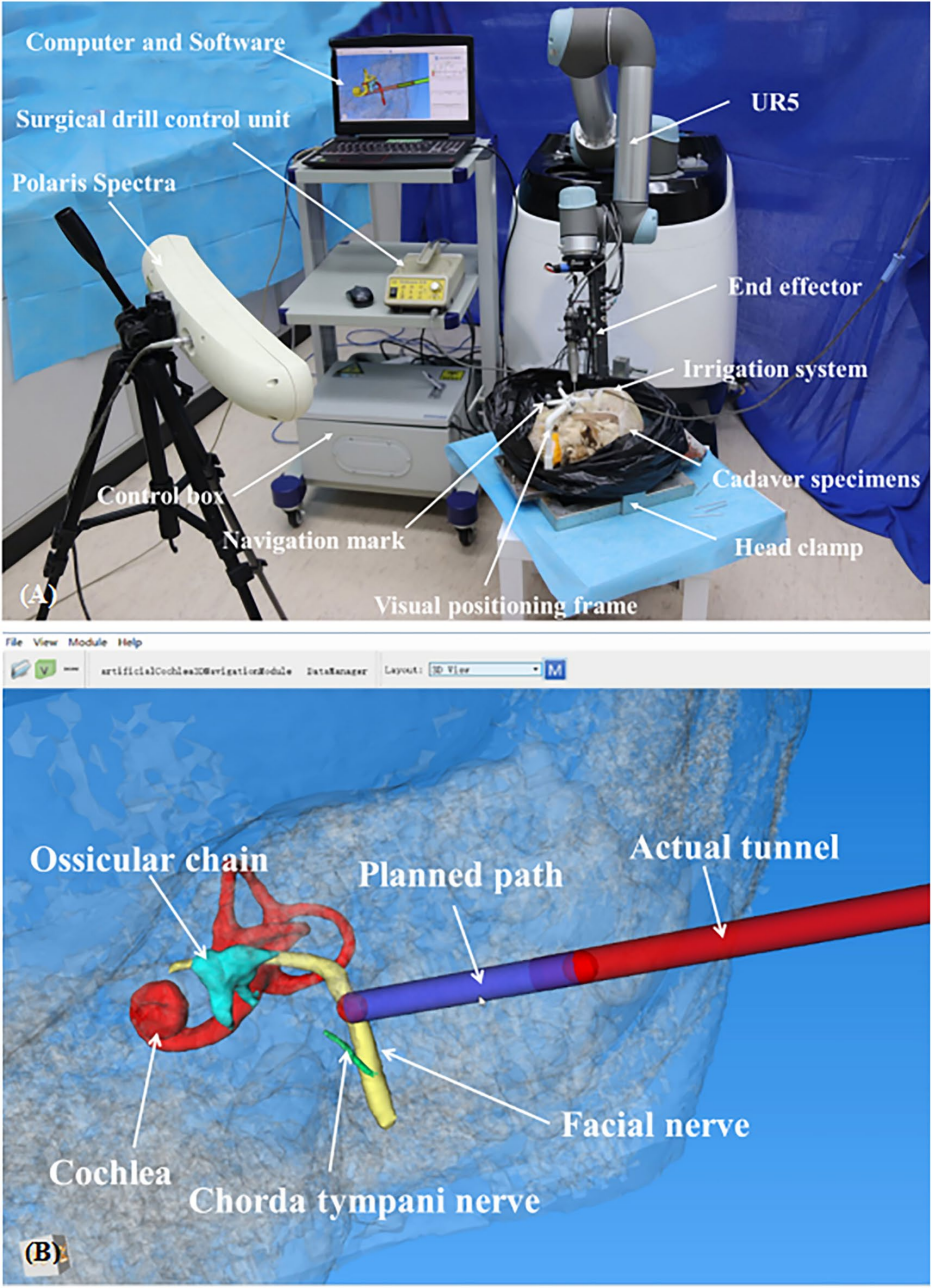
(**A**) The surgical robotic system includes the 6-DOF robotic arm, the end effector (1-DOT), the robot control system, the navigation tracking system, the computer and software system, the otological drill system, the irrigation system, etc. (**B**) Observe whether the drill bit advances along the planned path on the software system and terminate the operation in time if the error is too large.

Each specimen was fixed on a special head clamp, with the operating ear facing up, to prevent intraoperative shifting. Afterwards, the arm and hand-held drill power system (NSK Volvere GX, Tokyo, Japan) were mounted to the base of the robot and immobilized on the side close to the ear, 40–70 cm away from the head frame, so that the range of motion of the arm could completely cover the surgical area. Furthermore, the optical navigation system was installed on the opposite side of the robot maintained at a distance of 100–150 cm from the specimen, to ensure that the marks on the robot arm and the frame were included in the recognition field-of-view (Fig. 7).

The position of the drill bit (Medtronic, Minneapolis, MN, USA) was calibrated. Different drill bits were used for each of the 3 stages. A cutting drill bit (2.35-mm diameter, 70-mm length, 3.0-mm tip diameter) was used from the surface of the mastoid to the facial recess. A twist drill (2.35-mm diameter, 70-mm length, 1.8-mm tip diameter) was used to penetrate the facial recess. Then, a diamond drill bit (2.35-mm diameter, 70-mm length, 1.0-mm tip diameter) was used to enter the basal turn of the cochlea. After each drilling stage, the drill bit was returned from the same path and the other drill bit inserted for the next stage of drilling. The drilling speed for the first stage was 3000–4500 r/min, and that for the second stage and third stage was 1500–3000 r/min. Moreover, the advance speed of all drill bits was 0.04 mm/s, with continuous irrigation.

### Postoperative analysis

After the robot-assisted drilling, each cadaveric specimen again underwent CT at the Department of Radiology, Peking University Third Hospital, for a high-resolution scan (SOMATOM Definition Flash, Siemens; slice thickness = 0.4 mm, pitch = 0.2 mm). The CT data were introduced into Mimics 20.0 software, and using an axial view, it was observed whether the cochleostomy was located on the basal turn of the cochlea. The spatial coordinates of the skull surface entry point and the cochlear target point were recorded. Then, space fitting was performed in the software, using the 4 titanium screws as references for comparison of preoperative and postoperative images, and the errors between the actual entry/target point and the planned entry/target point were calculated. Simultaneously, we measured the closest distance from the margin of the tunnels to the edge of the facial nerves, ossicular chains, and posterior wall of the external acoustic meatus. In order to verify the effect of the drilling, all specimens were dissected after drilling.

### Ethical statement

This study was approved by the Peking University Third Hospital Medical Ethics Committee (IRB00006761-2,015,189). All cadavers were obtained from the Beijing Society for Anatomical sciences and every donors or next-of-kin signed and informed consent for the use of donors’ bodies after donors’ death for teaching and research purposes. All trials were performed in accordance with the principles of the Declaration of Helsinki.
